# Successful ATAC-Seq From Snap-Frozen Equine Tissues

**DOI:** 10.3389/fgene.2021.641788

**Published:** 2021-06-16

**Authors:** Sichong Peng, Rebecca Bellone, Jessica L. Petersen, Theodore S. Kalbfleisch, Carrie J. Finno

**Affiliations:** ^1^Department of Population Health and Reproduction, School of Veterinary Medicine, University of California, Davis, Davis, CA, United States; ^2^Veterinary Genetics Laboratory, School of Veterinary Medicine, University of California, Davis, Davis, CA, United States; ^3^Department of Animal Science, University of Nebraska-Lincoln, Lincoln, NE, United States; ^4^Department of Veterinary Science, Gluck Equine Research Center, University of Kentucky, Lexington, KY, United States

**Keywords:** FAANG, horse, cryopreserved, chromatin, epigenetics

## Abstract

An assay for transposase-accessible chromatin with high-throughput sequencing (ATAC-seq) has become an increasingly popular method to assess genome-wide chromatin accessibility in isolated nuclei from fresh tissues. However, many biobanks contain only snap-frozen tissue samples. While ATAC-seq has been applied to frozen brain tissues in human, its applicability in a wide variety of tissues in horse remains unclear. The Functional Annotation of Animal Genome (FAANG) project is an international collaboration aimed to provide high quality functional annotation of animal genomes. The equine FAANG initiative has generated a biobank of over 80 tissues from two reference female animals and experiments to begin to characterize tissue specificity of genome function for prioritized tissues have been performed. Due to the logistics of tissue collection and storage, extracting nuclei from a large number of tissues for ATAC-seq at the time of collection is not always practical. To assess the feasibility of using stored frozen tissues for ATAC-seq and to provide a guideline for the equine FAANG project, we compared ATAC-seq results from nuclei isolated from frozen tissue to cryopreserved nuclei (CN) isolated at the time of tissue harvest in liver, a highly cellular homogenous tissue, and lamina, a relatively acellular tissue unique to the horse. We identified 20,000–33,000 accessible chromatin regions in lamina and 22–61,000 in liver, with consistently more peaks identified using CN isolated at time of tissue collection. Our results suggest that frozen tissues are an acceptable substitute when CN are not available. For more challenging tissues such as lamina, nuclei extraction at the time of tissue collection is still preferred for optimal results. Therefore, tissue type and accessibility to intact nuclei should be considered when designing ATAC-seq experiments.

## Introduction

The completion of the equine genome assembly (Wade et al., 2009; Kalbfleisch et al., 2018) has enabled research leading to novel discoveries concerning the health and reproduction of horses (Finno and Bannasch, 2014; Ghosh et al., 2018; Raudsepp et al., 2019). However, despite having the same genomic sequence, differential regulation of gene expression leads to tissue-specific profiles. A lack of understanding of gene regulation has largely stalled research of complex traits in horses. In humans and mice, the Encyclopedia of DNA Elements (ENCODE) project has provided an abundance of data for understanding gene regulation and its role in complex diseases and traits (Qu and Fang, 2013). Unfortunately, limited resources are currently available in the horse. The Functional Annotation of Animal Genome (FAANG) initiative (The FAANG Consortium et al., 2015) is an international collaboration aimed to bridge this gap between genotype and phenotype. The equine FAANG project has successfully generated a biobank of over 80 tissues and bodily fluids of two reference animals (Burns et al., 2018). RNA-seq of 32 tissues (unpublished, data access: PRJEB26787), as well as the identification of tissue specific histone marks for eight prioritized tissues (Kingsley et al., 2019), from this biobank has been performed. Additional projects are underway to identify tissue specific chromatin states to integrate all of these datasets and build a robust tissue specific functional annotation atlas in the horse (Giuffra et al., 2019).

An important component of gene expression and regulation is chromatin accessibility. Active genes and regulatory elements are typically found within or near regions of the DNA accessible to transcription factors. Therefore, identifying open chromatin regions is a crucial step to identify and categorize tissue specific regulatory elements in order to advance our understanding of complex traits in the horse. An assay for transposase-accessible chromatin with high-throughput sequencing (ATAC-seq) (Buenrostro et al., 2015) is commonly used to identify regions of open chromatin. A typical ATAC-seq protocol requires nuclei extracted from fresh tissues. Halstead et al. (2020b) proposed a modified ATAC-seq protocol to allow long-term storage of cryopreserved nuclei (CN) extracted from fresh tissues. Still, the intensive efforts needed to prepare and cryopreserve nuclei during a large-scale tissue collection prove to be difficult. Alternatively, Corces et al. (2017) successfully applied a modified ATAC-seq (Omni-ATAC) protocol on frozen human brain tissues. However, the applicability of Omni-ATAC has not been tested in a wide variety of tissues in horse where nuclei extraction may prove challenging. Additionally, it has been shown that in cultured cells cryopreservation is preferable to flash-freezing process in order to preserve native chromatin structures (Milani et al., 2016). To our knowledge, no studies have investigated the effect of snap freezing on tissues for ATAC-seq library generation in comparison to CN preps. Additionally, the library preparation step is a major source of variation in RNA-seq studies (McIntyre et al., 2011), particularly at low read depth. As a result, RNA-seq data generated from different laboratories or at different times cannot often be directly compared. For a collaborative project, it is important to assess the effect of technical variations to better inform project planning and analytical decisions for data integration.

To address these gaps of knowledge in the applicability of ATAC-seq in snap-frozen horse tissues, and to provide a guide for future ATAC-seq studies to assess chromatin accessibility, we compared data from CN prepared from fresh tissue to that of nuclei extracted from snap-frozen tissues collected from the two mares from the initial equine FAANG biobank study (Burns et al., 2018). In order for this comparison to be informative and applicable to a wide range of tissues, we utilized both liver, a highly cellular and homogenous tissue type, and lamina, a relatively acellular tissue unique to the horse. Equine laminae are highly vascularized interdigitated dermal and epidermal tissues in the equine foot that form the attachment between the hoof wall and the third phalanx. Inflammation of laminae in horses (i.e., laminitis) is a devastating disease that impacts many breeds of horses and often leads to euthanasia. Therefore, gene regulation in laminae is of particular interest to equine geneticists and veterinary practitioners as this debilitating and life-threatening disease estimated to impact up to 34% of the horse population (Wylie et al., 2011). Laminitis is also the primary clinical consequence of equine metabolic syndrome (EMS) (Durham et al., 2019). EMS is a complex syndrome that requires constant veterinarian care and diet control, impacting an estimated 18 to 27 percent of horse population (Durham et al., 2019). Liver is the primary metabolic organ with a homogenously cellular structure. Detailed knowledge of gene expressions and regulations in healthy liver provides a baseline for studying impaired metabolism in horses with EMS. Additionally, to assess the effect of library preparation techniques, snap-frozen tissues and CN from this pilot study were sent to two different core laboratories for library generation and subsequent sequencing. We hypothesized that (1) ATAC-seq using frozen tissues would identify comparable peaks to those using CN from fresh tissues, (2) libraries generated from liver will have better quality than those from laminae, and (3) similar to what was found in RNA-seq studies there will be a significant amount of variation between the libraries generated by two laboratories.

## Materials and Methods

### Tissue Collection and Nuclei Isolation

Liver and lamina tissues from two mares (AH2 and AH1) were collected as described in Burns et al. (2018). Briefly, two healthy adult Thoroughbred mares (AH1: 5 years old; AH2: 4 years old) were closely examined by veterinarians prior to tissue collection. Nuclei were isolated from liver and lamina tissues immediately following tissue collection and cryopreserved following protocols published in Halstead et al. (2020a) with some modifications for lamina. Briefly, additional incubation periods with collagenase were added to assist in homogenization (see Supplementary Material). These are referred to as CN. Additionally, at time of collection, approximately 1 g aliquots of tissue were snap frozen in liquid nitrogen for nuclei extraction at a later time. These are referred to as frozen tissue-derived nuclei (FTDN).

### ATAC-Seq Library Preparation and Sequencing

Both snap frozen tissues and CN were stored at −80°C for 3 years until shipped on dry ice overnight to two commercial laboratories (L1 and L2) for library preparation. Nuclei were extracted from frozen tissues using each laboratory’s internally optimized protocol (see Supplementary Material). Extracted Nuclei (FTDN) and CN were used to prepare ATAC libraries (Supplementary Methods and Supplementary Table 1). Libraries were sequenced on an Illumina HiSeq 4000, paired-end 2 × 75 bp (L1) or NextSeq 500, paired-end 2 × 42 bp (L2) with a targeted depth of 30 million read pairs.

### ATAC-Seq Data Analysis

Read QC was carried out using FastQC (Andrews, 2010). Adapters and low-quality ends were trimmed using TrimGalore (Krueger, 2019) and Cutadapt (Martin, 2011). Reads were then aligned to reference genome EquCab3 using BWA-MEM algorithm from BWA (Li and Durbin, 2009) using default parameters. Post-alignment filtering was employed to remove low mapping quality reads, mitochondrial reads, and PCR duplicates using Samtools (Li et al., 2009) and Sambamba (Tarasov et al., 2015). Genome coverage was analyzed using deepTools (Ramírez et al., 2016). Specifically, bamCoverage was used to convert bam files to bigwig files, using RPKM to normalize coverage with exact scaling (–normalizeUsing RPKM –exactScaling). Then multibigwigSummary was used to calculate average coverage across 1,000 bp windows (-bs 1,000). plotPCA was used to calculate eigen values based on all genomic windows (–ntop 0) and top 2 principle components were plotted using matplotlib (Caswell et al., 2020). Custom scripts were used to analyze sample correlation, clustering, and correlation with ChIP-seq data and annotated genes using Python packages numpy (Harris et al., 2020), scipy (SciPy 1.0 Contributors et al., 2020), pandas (Reback et al., 2020), and matplotlib (Caswell et al., 2020). Open regions were identified using HMMRATAC (–threshold 2 –score fc -u 20 -l 10) (Tarbell and Liu, 2019) and MACS2 (-q 0.05 -B –broad -f BAMPE) (Zhang et al., 2008). Jaccard indices were calculated using pybedtools (Quinlan and Hall, 2010; Dale et al., 2011)Quinlan and Hall, 2010) for each pair of biologic replicates with default parameters. More detailed pipeline is available at https://github.com/SichongP/FAANG_ATACseq.

### Histone ChIP-Seq Data Processing

Histone ChIP-seq data were downloaded from FAANG data repository^1^ under accession PRJEB35307. Histone marks were determined according to Kingsley et al. (2019) and compared with open chromatin regions analyzed in this study for both liver and lamina.

### ATAC-Seq Peak Validation With Histone Marks

ATAC-seq peaks called by HMMRATAC and MACS2 were validated using histone ChIP-seq data following (Tarbell and Liu, 2019) with modifications to utilize available data in the horse. First, the following sets of peaks were generated from Kingsley et al. (2019) data:

Real positive set (RP): peaks from either H3K4me1 or H3K4me3 that overlap H3K27ac peaks
Real negative set (RN): peaks from H3K27me3 data

Then, following metrics were calculated for each dataset:

T⁢P=n⁢u⁢m⁢b⁢e⁢r⁢o⁢f⁢b⁢a⁢s⁢e⁢s⁢i⁢n⁢c⁢a⁢l⁢l⁢e⁢d

A⁢T⁢A⁢C-s⁢e⁢q⁢p⁢e⁢a⁢k⁢s⁢o⁢v⁢e⁢r⁢l⁢a⁢p⁢p⁢i⁢n⁢g⁢R⁢P

F⁢P=n⁢u⁢m⁢b⁢e⁢r⁢o⁢f⁢b⁢a⁢s⁢e⁢s⁢i⁢n⁢c⁢a⁢l⁢l⁢e⁢d

A⁢T⁢A⁢C-s⁢e⁢q⁢p⁢e⁢a⁢k⁢s⁢o⁢v⁢e⁢r⁢l⁢a⁢p⁢p⁢i⁢n⁢g⁢R⁢N

$$P\,r\,e\,c\,i\,s\,i\,o\,n=\frac{T\,P}{T\,P+F\,P}$$

$$R\,e\,c\,a\,l\,l=\frac{T\,P}{R\,P}$$

$$F\,a\,l\,s\,e\,P\,o\,s\,i\,t\,i\,v\,e\,R\,a\,t\,e\,\left(F\,P\,R\right)=\frac{F\,P}{R\,N}$$

Increasing quality scores as produced by MACS2 or HMMRATAC were used as the cutoff score to filter peaks before the remaining peaks were used to calculate above metrics. Changes in the metrics as the cutoff score increased were used to identify the thresholds at which to filter final sets of open chromatin peaks.

### RNA-Seq Data Processing

RNA-seq reads from liver and lamina of the same two animals were available from a separate project under European Nucleotide Archive accession PRJEB26787. Briefly, RNA was isolated from liver or lamina tissues using Trizol chloroform phase separation followed by a column cleanup using Zymo Research Direct-Zol Mini columns. TruSeq mRNA libraries were prepared at Minnesota Genomics Center (Minneapolis, MN, United States) and sequenced at 125 bp paired-end. These reads were quantified against Equcab3 Ensembl annotated genes (Kalbfleisch et al., 2018; Cunningham et al., 2019) using Salmon (Patro et al., 2017) mapping-based mode. Transcript level counts were aggregated into gene level using the R package tximport (Soneson et al., 2015) and final counts were normalized using the variance-stabilizing transformation method from DESeq2 vst function (Love et al., 2014).

### ATAC-Seq Peak Validation With RNA-Seq Data

Ensembl annotated genes were classified as open or closed depending on whether their presumed promoter regions (1 kb upstream of annotated gene start) overlapped with identified ATAC-seq peaks. These genes were then compared to their RNA abundance estimated using FAANG data.

## Results

**FIGURE 1:**
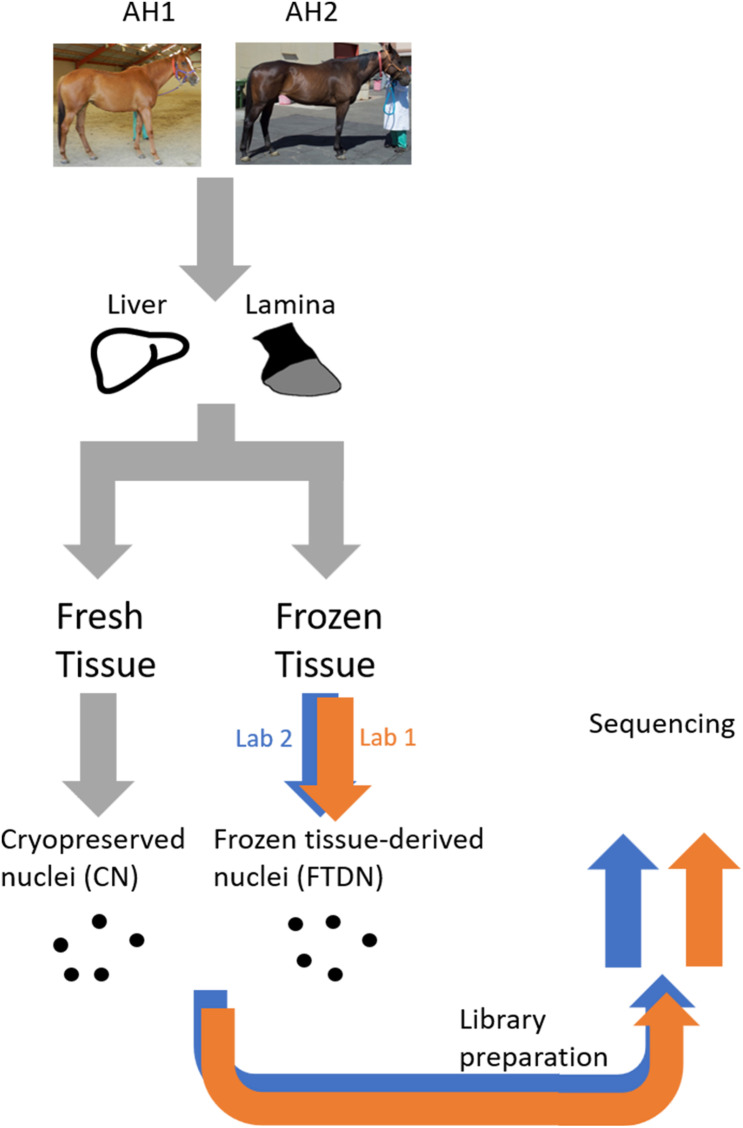
A schematic of the experimental design. All samples were prepared at UC Davis prior to shipment to the core laboratories. Samples used were obtained from an equine biobank of two horses (AH1 and AH2), as previously described (Burns et al., 2018).

Libraries prepared by two laboratories (L1 and L2) using nuclei isolated from snap-frozen tissues (FTDN) or cryopreserved from tissues at time of collection (CN) from liver and lamina of two animals (AH1 and AH2, Thoroughbred adult mares) were sequenced at PE75 on an Illumina HiSeq 4000 (L1) or PE42 on an Illumina NextSeq 500 (L2). Figure 1 shows a schematic of the experimental design.

### Library Fragmentation

ATAC-seq libraries are expected to present a laddering pattern that corresponds to different nucleosome-bound fragments. Supplementary Figures 1, 2 show fragment size distributions of ATAC libraries as determined by sequencing and Agilent Fragment Analyzer (L1) or TapeStation (L2) from L1 and L2, respectively. In general, liver libraries showed distinguishable laddering pattern while in lamina libraries, only the fragment size corresponding to nucleosome-free fragments was observed.

**FIGURE 2:**
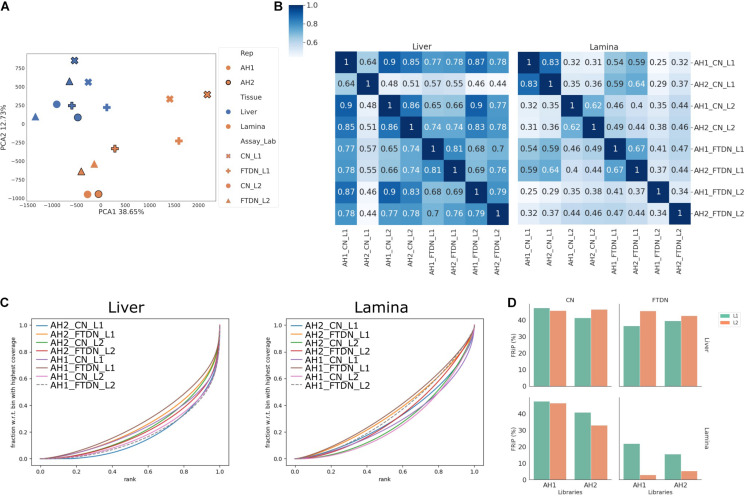
Read coverage correlation between libraries. Read depth was normalized across all libraries. **(A)** Principal component analysis of genome coverage, showing the first two principal components. **(B)** Pearson correlation of genome coverage in liver (left) and lamina (right) libraries. Linkage was calculated using Farthest Point Algorithm. **(C)** Fingerprint plot of genome coverage in liver (left) and lamina (right) libraries. **(D)** Enrichment as measured by FRiP in each library.

### Sequencing Read Lengths

Since libraries from L1 and L2 were sequenced at different lengths (75 and 42 bp, respectively), we trimmed longer reads from L1 from 3′ down to 42 bp and compared read alignment statistics to those obtained using full length reads (75 bp), after appropriate quality trimming. There were no significant changes in read alignment statistics, with less than 0.02% fewer reads aligned and less than 0.3% fewer reads identified as duplicates for each library after length trimming. Therefore, we proceeded with data analysis using original full length reads from both laboratories.

### Duplication Rate and Mitochondrial Contamination

Overall, liver libraries have higher mitochondrial contamination than lamina libraries, likely due to higher metabolic activities in liver (Supplementary Figure 3A). Among liver samples, CN libraries prepared by L1 contained 56 and 81% duplicates, with 37 and 23% mitochondrial reads in AH1 and AH2, respectively. In comparison, the CN libraries from L2 contained 31 and 24% duplicates, with 23 and 10% mitochondrial reads from AH1 and AH2, respectively, (Supplementary Figure 3A). It was suspected that the higher amount of mitochondrial contamination contributed to the higher duplication rate and led to lower library complexity. To test this hypothesis, resequencing was performed for the liver CN libraries from L1. The number of unique nuclear reads from AH2 largely remained unchanged despite increasing read depth three-fold. For AH1, however, twice the number of unique nuclear reads was obtained after the total read depth was increased (Supplementary Figure 3B). Both the fingerprint plot and fraction of reads in peaks (FRiP) identified a decrease in enrichment for AH1 with increased sequencing depth but little change for AH2 (Supplementary Figure 3C and Supplementary Table 2). This suggests that, in the AH1 library, while further sequencing increased the number of unique reads, it did not substantially improve peak detection. Lowered enrichment in the resequenced AH1 library suggests that a majority of additional unique reads are less enriched background reads. In the AH2 library, however, resequencing did not significantly improve library complexity, due to more cycles of amplification during library preparation and therefore, higher PCR duplication rate in the library.

### Genome Coverage and Enrichment

To assess which part of the ATAC-seq protocol contributed more to library variations and complexities, we compared genome coverage and enrichment (Figure 2). Principle component analysis (PCA) revealed that liver libraries generally clustered closely together, while more variation was observed for the lamina libraries (Figure 2A). Within the lamina libraries, there is a clear clustering based on which laboratory prepared the libraries. The lamina libraries from L2 clustered closely with each other and with liver libraries while the lamina libraries from L1 clustered further away from liver libraries (Figure 2A). Heatmaps of the genome coverage Pearson correlation showed that liver CN libraries yielded well-correlated results, with the exception of that from AH2 by L1 (Figure 2B). This is consistent with low complexity of that library shown in Supplementary Figure 3. On the other hand, little correlation is observed among lamina library preparations (Figure 2B). Since no input libraries were used for ATAC-seq experiments (Buenrostro et al., 2015), synthetic Jensen-Shannon distance (SJSD) was used, together with Area Under Curve (AUC) from fingerprint plots, to assess the enrichment of each library (Figure 2C and Supplementary Table 3). In general, liver libraries showed higher enrichment than lamina libraries. Within liver libraries, CN libraries were more enriched than FTDN libraries from L1, while both libraries from L2 showed similar enrichment. Within lamina libraries, both laboratories generated more enriched libraries from CN than from FTDN. This is further exemplified in Figure 2D, showing the FRiP in each library.

### Peak Calling

**FIGURE 3:**
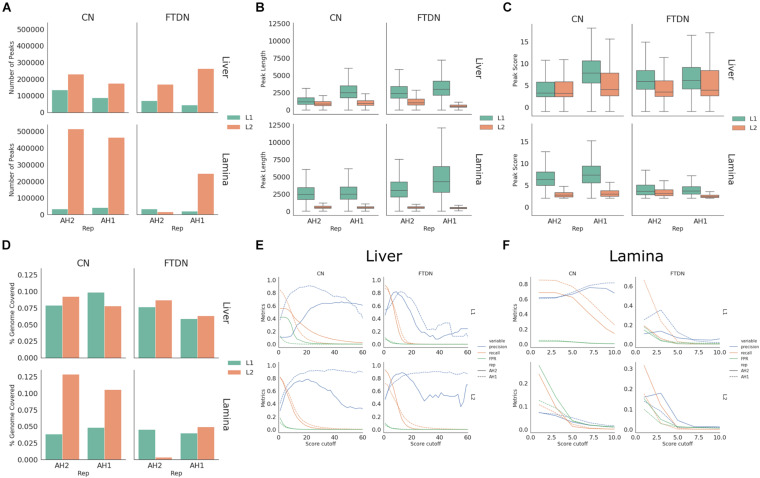
HMMRATAC peak calling statistics. **(A)** Number of peaks, **(B)** peak length distribution, **(C)** peak score distribution, and **(D)** percent of genome covered by peaks for each library. **(E,F)** Peak metrics assessed using ChIP-seq dataset in liver **(E)** and lamina **(F)** libraries.

To identify accessible chromatin regions, MACS2 (Zhang et al., 2008) and HMMRATAC (Tarbell and Liu, 2019) were used to call peaks and results from both programs were compared. To control for sequencing depth, all libraries were down-sampled to 60 million unique reads that are suitable for peak calling using sambamba view function. Using MACS2 (-q 0.05 -B–broad -f BAMPE), 31,000–721,000 peaks were identified. While using HMMRATAC (–threshold 2 –score fc -u 20 -l 10), 14,000–514,000 peaks were identified. Overall, using HMMRATAC, peaks identified from lamina libraries had lower quality [fewer (Figure 3A) and shorter peaks (Figure 3B) with lower scores (Figure 3C)] than those from liver libraries. For liver libraries, CN generated comparable results to FTDN while, in lamina libraries, CN outperformed FTDN (Figure 3D). Similar results were obtained when peaks were called using MACS2 (Supplementary Figures 4A,D).

To better assess the quality of peaks, we used histone mark ChIP-seq data generated from the same samples as described in Kingsley et al. (2019). A set of metrics, precision, recall, and false positive rate (FPR), were generated for different cutoff scores as described in Methods. These metrics were then plotted against cutoff scores. Consistent with the observation of peak lengths and scores, peaks called using HMMRATAC from liver libraries had higher precision and recall rates and lower false positive rates (Figure 3E) than lamina (Figure 3F). Consistent with observations of library quality, CN liver libraries of AH2 from L1 have lower recall and precision rates than that from L2 or that of AH1, despite having same unique read depth (Figure 3E). Comparing peaks identified by two programs, HMMRATAC identified peaks with higher recall and precision rates than MACS2 (Supplementary Figures 4E,F).

### ATAC-Seq Peak Validation

Despite higher quality from L2 in liver AH2 CN library, L1 produced the only libraries from laminae with high quality peaks (Figure 3F). Therefore, to maximize usable data, libraries from L1 were chosen for all further analyses. HMMRATAC was used as it produced generally better metrics and because it allowed interrogation of nucleosome-bound regions vs. nucleosome-free regions for future studies.

**FIGURE 4:**
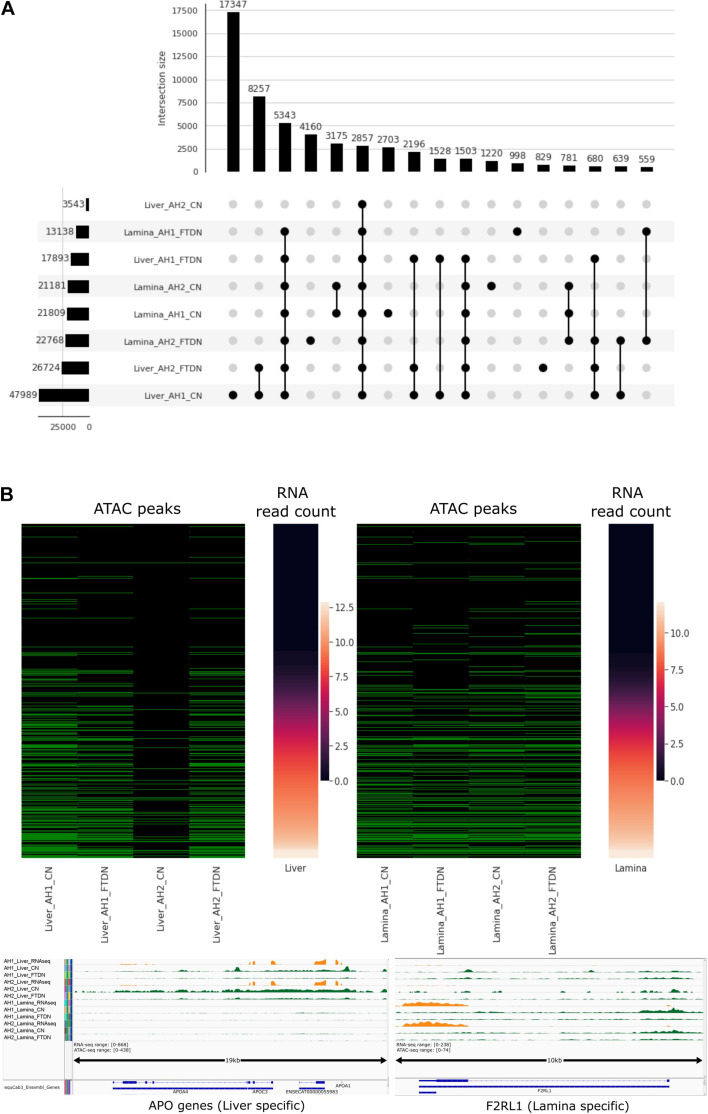
Filtered ATAC-seq peaks. **(A)** Intersection plot of quality filtered peaks from each library. Bottom left panel shows filtered peak count in each library; bottom right panel shows different intersections (BedTools, 1 bp minimum) of peaks where filled dots indicate presence of peaks in corresponding library; Top panel shows peak count in each intersection. **(B)** Relationship between promoter accessibility and gene expression (mean vst transformed count) in liver (top left) and lamina (top right). Green cell in ATAC peaks indicate presence of ATAC peaks and black cells indicate absence. Bottom panel shows bigwig tracks of RNA-seq and ATAC-seq read abundance (normalized using RPKM)near *APO genes* (left, liver specific) and *F2RL1* (right, lamina specific) transcription start sites.

A cutoff score, where the precision and recall lines intercept, was used for each sample set to filter peaks identified by HMMRATAC. Final peak counts are shown in Table 1. Consistent with previous observations, liver samples generated the most high-quality peaks, while CN libraries outperformed FTDN libraries. Using UpSetPlot (Nothman, 2020) based on (Lex et al., 2014), we identified overlapping peaks in each dataset (Figure 4A). AH1 liver CN library generated the most unique peaks, consistent with the previous observation that this library has highest library complexity. Since 17,347 unique peaks were identified from this library only, a precision score of these unique peaks was calculated using histone ChIP-seq data mentioned above. A precision score of 18.4% was observed in these peaks, suggesting a high rate of false positive peaks. This further highlights the importance of replicates in an ATAC-seq experiment. FTDN libraries did not yield significant number of unique peaks that were not detected in CN libraries. Despite a relatively low quality of the lamina libraries, 12,256 unique peaks were detected from the lamina libraries.

**TABLE 1:** Cutoff used to filter peaks and metrics of filtered peaks.

Tissue	Rep	Nuclei prep	Cutoff score	Count	AvePeakLen	MedianPeakLen	Bases covered	Confirmed count	Jaccard index
Liver	AH1	CN	6	61,473	2,937.0	2,600	180,547,240	3,646	0.05
Liver	AH2	CN	16	3,810	2,428.8	2,090	9,253,670		
Liver	AH1	FTDN	6	22,588	3,701.6	3,300	83,611,751	18,596	0.35
Liver	AH2	FTDN	6	33,782	3,059.1	2,650	103,343,612		
Lamina	AH1	CN	6	28,418	3,106.6	2,650	88,284,203	23,439	0.51
Lamina	AH2	CN	4	30,906	2,883.5	2,480	89,117,724		
Lamina	AH1	FTDN	2	19,886	5,061.4	4,300	100,651,092	17,619	0.35
Lamina	AH2	FTDN	2	33,762	3,361.9	3,010	113,504,835		

*Filtered peaks and their corresponding cutoff scores in each library. AvePeakLen, Average peak length; MedianPeakLen, Median peak length; Bases covered, number of bases covered by all peaks in a library; Confirmed count, overlapping peaks in both biological replicates; Jaccard index, jaccard index of two biological replicates.*

As an *in silico* validation of the results, peaks were overlapped with Ensembl gene annotation for EquCab3 (Kalbfleisch et al., 2018) at promoter regions (1 kb upstream of annotated gene start) to classify each promoter as open or closed. These classified promoter regions were then compared to RNA abundance at the corresponding gene level (Figure 4B). In liver, AH1 CN identified more open promoters where RNA expression levels are high but the results from the two assays (CN and FTDN) were highly comparable for this sample in liver. Fewer peaks were identified from AH2 CN, due to low library quality and issues in repeat freeze thaw cycles as outlined in the discussion. In lamina, CN assays identified more open promoters than FTDN. Manual inspection of some highly abundant genes in liver and laminae validate accurate identification of open chromatin in each tissue (Figure 4B).

Overall, our results confirm that extracting nuclei from snap-frozen tissues for ATAC-seq library preparations negatively affects the library quality, resulting in fewer peaks detected. However, when CN from freshly collected tissue are not available, these data show that snap-frozen tissues can be used to prepare ATAC-seq libraries to give reliable peak calls, with the caveat that some regions of open chromatin will be missed. However, results from laminae suggest that for more challenging tissue types, fresh tissue extraction is a requirement.

## Discussion

In this pilot study, we compared two tissues (liver and laminae, representing homogenous cellular and relatively acellular nuclei extraction, respectively) from the equine FAANG project for ATAC-seq library generation, using two nuclei extraction methods. Nuclei extracted and cryopreserved immediately after tissue collection and nuclei isolated from snap-frozen tissues were used to determine suitable methods for performing ATAC-seq to identify accessible chromatin regions in a wide variety of equine tissues for functional annotation. Similar to what was identified by Halstead et al. (2020a), we determined that ATAC-seq can be used to characterize open chromatin in animal tissue but optimization is necessary to have a robust data set across tissues. Further, we found that while CN generally yield more peaks, frozen tissues can still be used to isolate nuclei and identify accessible regions. However, the quality of libraries generated by the frozen tissue protocol suffered when nuclei were extracted from a more challenging, relatively acellular tissue, such as laminae. Therefore, for challenging tissues, care should be taken at time of collection to prioritize those tissues for nuclei extraction and cryopreservation when possible.

We also showed that the frozen tissue protocol is more prone to variations introduced at the library preparation step. Specifically, FTDN liver libraries generated at two different laboratories only have a moderate correlation (0.68 for AH1 and 0.76 for AH2). Our analysis suggests that, similar to RNA-seq experiments, library preparation can introduce large variation that will impact subsequent data quality, specifically peak detection for ATAC-seq studies. However, since the two commercial laboratories used different internally optimized protocols, it is impossible to determine whether the variation was protocol-specific or lab-specific. Nonetheless, it is advisable for all ATAC-seq library preparations to be performed at a single site using the same protocols to minimize variability in datasets when trying to integrate information.

During library preparation, the CN aliquot from AH2 was partially thawed twice by L1 (first for an optimization experiment (data not shown) and then a second time to perform the data collection). The nuclei obtained during the second partial thawing were used in this study. Due to the precipitation of nuclei and contaminating mitochondria, this was likely the cause of low quality observed in that library preparation. The effect of different read lengths used by two laboratories was investigated and deemed to have no significant impact on read alignment. Our analysis suggested a detrimental impact on data quality by this practice and resequencing of this particular library also did not improve data quality nor was this resequencing effort able to identify more peaks. Therefore, it is advisable to avoid repeated partial thawing of CN aliquots.

Library fragment size screening using gel electrophoresis proved to be predictive of final fragment size distribution in sequencing results and data quality. As indicated in Supplementary Figures 1, 2, a strong signature corresponding to nucleosome-free fragments without accompanying signatures for nucleosome-bound regions does not necessarily mean a high enrichment of nucleosome-free fragments. It could also indicate high levels of mitochondria contamination or fragmentation of chromatins before tagmentation, which are likely the cases in lamina libraries from L2.

We identified 20–33,000 accessible chromatin regions in lamina and 22–61,000 in liver, largely in line with observations of liver ATAC-seq from studies in other species (Ackermann et al., 2016; Foissac et al., 2019; Liu et al., 2019; Halstead et al., 2020b). As a preliminary study, we opted to include laboratory replicates in lieu of technical replicates in order to assess the effect of technical variations introduced during the library preparation step. Technical replicates would allow further validation of tissue specific open-chromatin. Following ENCODE standard (Landt et al., 2012) for ChIP-seq experiments, two biological replicates were collected for the FAANG project. However, more replicates would have allowed a more robust comparison between different protocols.

In this study, we demonstrated the feasibility of using snap-frozen tissues for ATAC-seq experiments for the equine FAANG project. For acellular tissues, more optimization is required for ATAC-seq experiments. We also showed that significant variation can be introduced during library preparation. This study provides important guidelines for planning future ATAC-seq experiments using equine FAANG tissues. We will use the guidelines established here to conduct ATAC-seq experiments on six other prioritized tissues in the mares. Furthermore, following these guidelines should enable the most meaningful integration of datasets across studies thus building a reliable functional tissue specific atlas of the equine genome which would advance our understanding of complex traits in the horse.

## Data Availability Statement

ATAC-seq data used in this study are available from the European Nucleotide Archive under the accession PRJEB41317. RNA-seq data from the liver and lamina tissues of the same two animals used in this study can be found from the European Nucleotide Archive under the accession PRJEB26787.

## Author Contributions

CF and SP were responsible for the conceptualization, methodology, investigation, formal analysis, resources, and writing of the manuscript. CF, RB, JP, and TK were responsible for the funding acquisition. All authors have reviewed the final manuscript.

## Conflict of Interest

The cost of library preparation and sequencing was partially covered by two core laboratories as part of collaboration to optimize ATAC-seq protocol using horse tissues.

## Footnotes

1 https://data.faang.org/home

## Supplementary Material

Supplementary Figure 1 Fragment size distributions of libraries from L1 as determined by sequencing and Fragment Analyzer.

Supplementary Figure 2 Fragment size distributions of libraries from L2 as determined by sequencing and tapestation.

Supplementary Figure 3 Duplication and mitochondrial contamination rates. **(A)** Total, mitochondrial, and unique nuclear read counts of all libraries; **(B)** Comparison between first sequencing run (left) and combined reads (right) from L1 liver CN libraries; **(C)** Fingerprint plot of L1 CN liver libraries.

Supplementary Figure 4 MACS2 peak calling statistics. **(A)** Number of peaks, **(B)** peak length distribution, **(C)** peak score distribution, and **(D)** percent of genome covered by peaks for each library. **(E,F)** Peak metrics assessed using ChIP-seq dataset in liver **(E)** and lamina **(F)** libraries.
