# Supplementary figures and images for: Successful ATAC-Seq From Snap-Frozen Equine Tissues
Source: Front Genet. 2021 Jun 16;12:641788. doi: 10.3389/fgene.2021.641788 (PMC8242358; doi:10.3389/fgene.2021.641788)

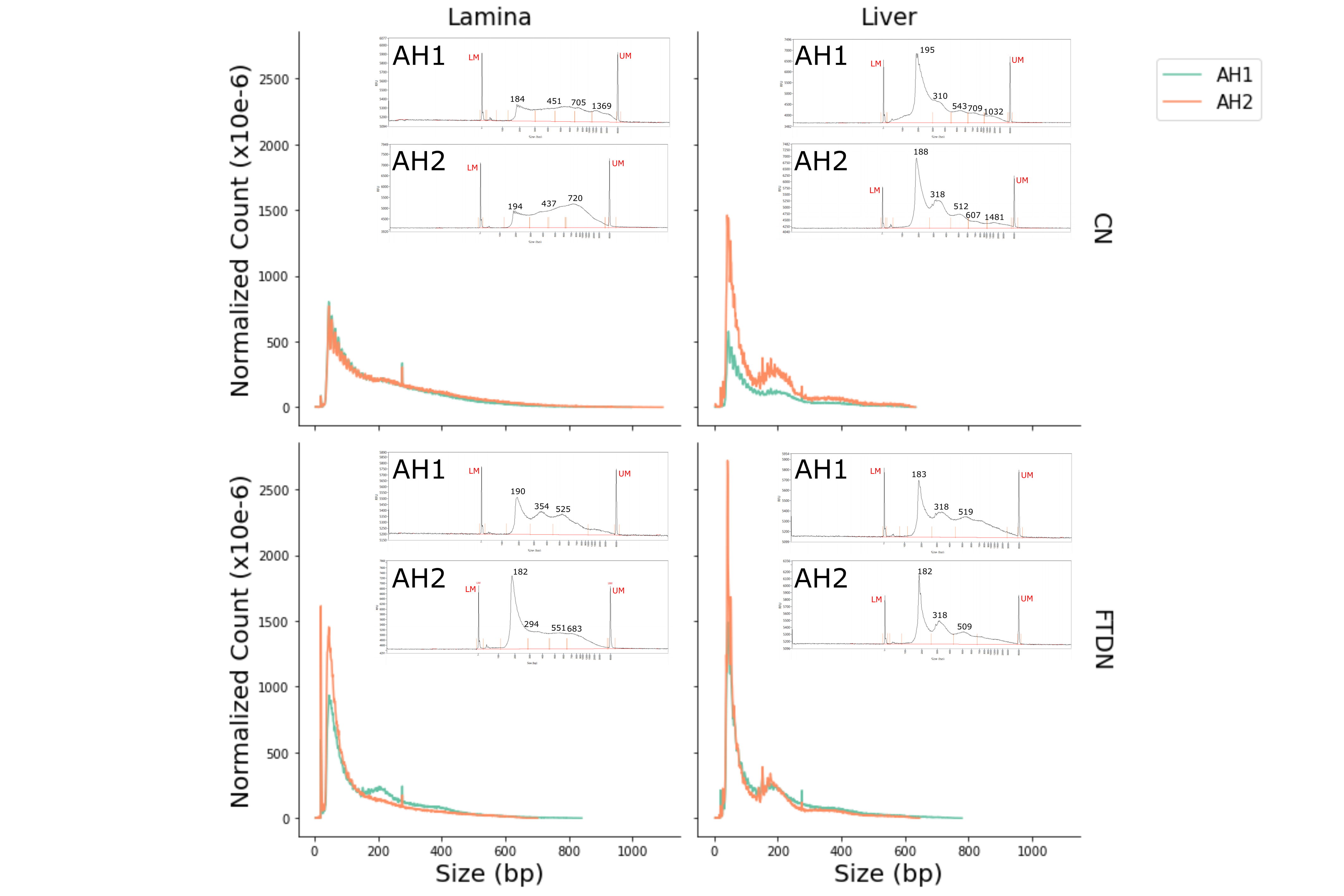

Supplement: Supplementary Figure 1 — Fragment size distributions of libraries from L1 as determined by sequencing and Fragment Analyzer. [file Image_1.TIF]

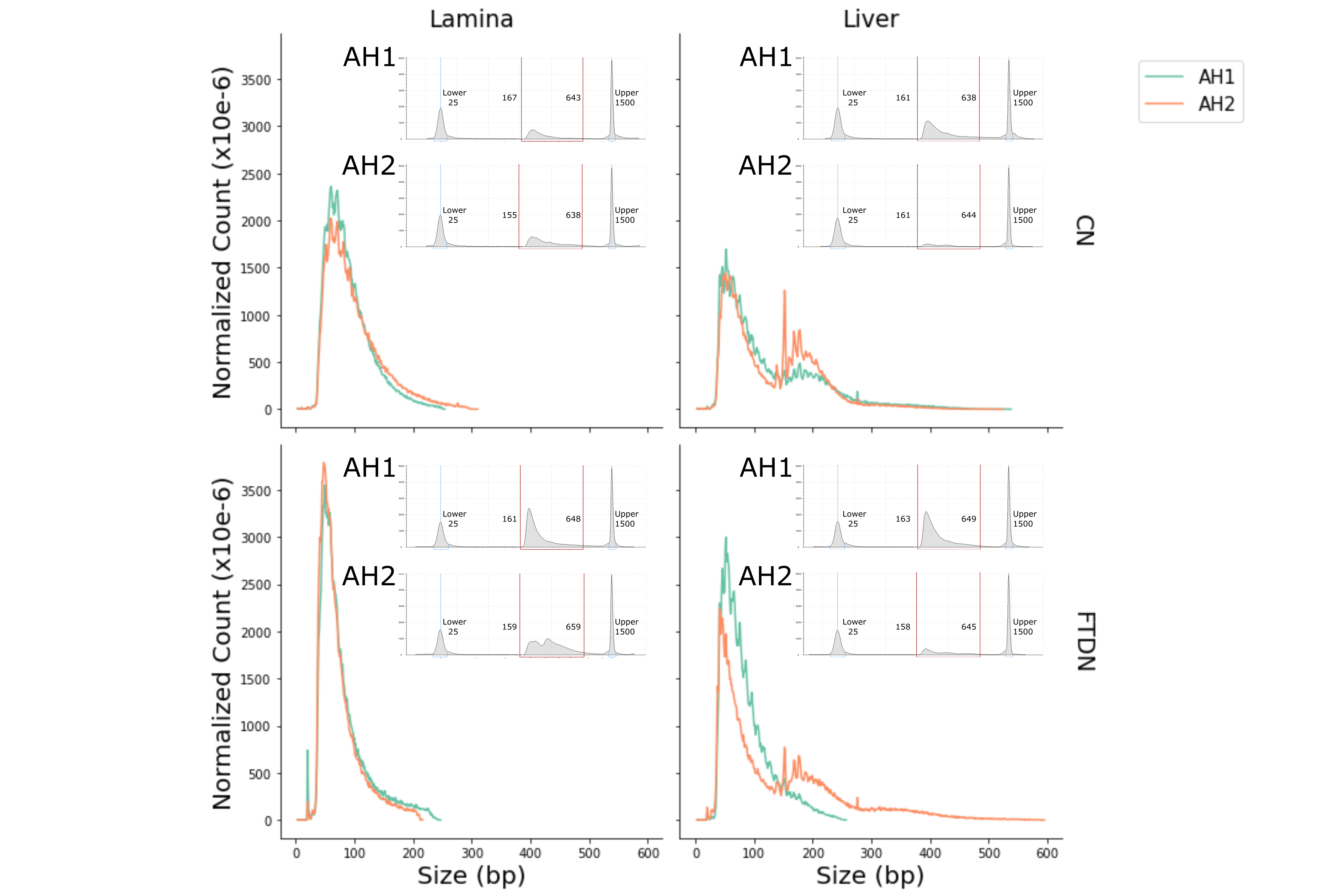

Supplement: Supplementary Figure 2 — Fragment size distributions of libraries from L2 as determined by sequencing and tapestation. [file Image_2.TIF]

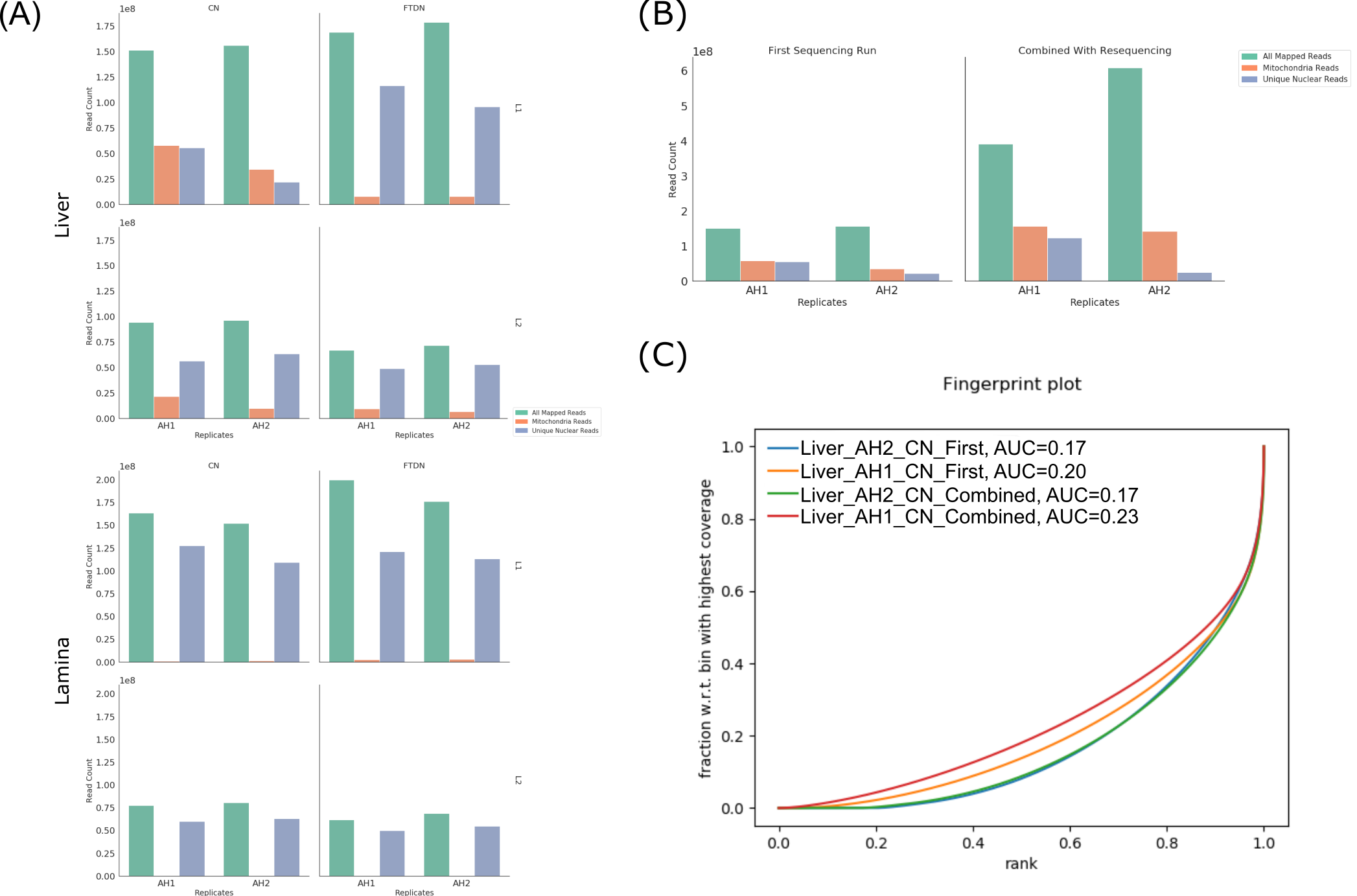

Supplement: Supplementary Figure 3 — Duplication and mitochondrial contamination rates. (A) Total, mitochondrial, and unique nuclear read counts of all libraries; (B) Comparison between first sequencing run (left) and combined reads (right) from L1 liver CN libraries; (C) Fingerprint plot of L1 CN liver libraries. [file Image_3.TIF]

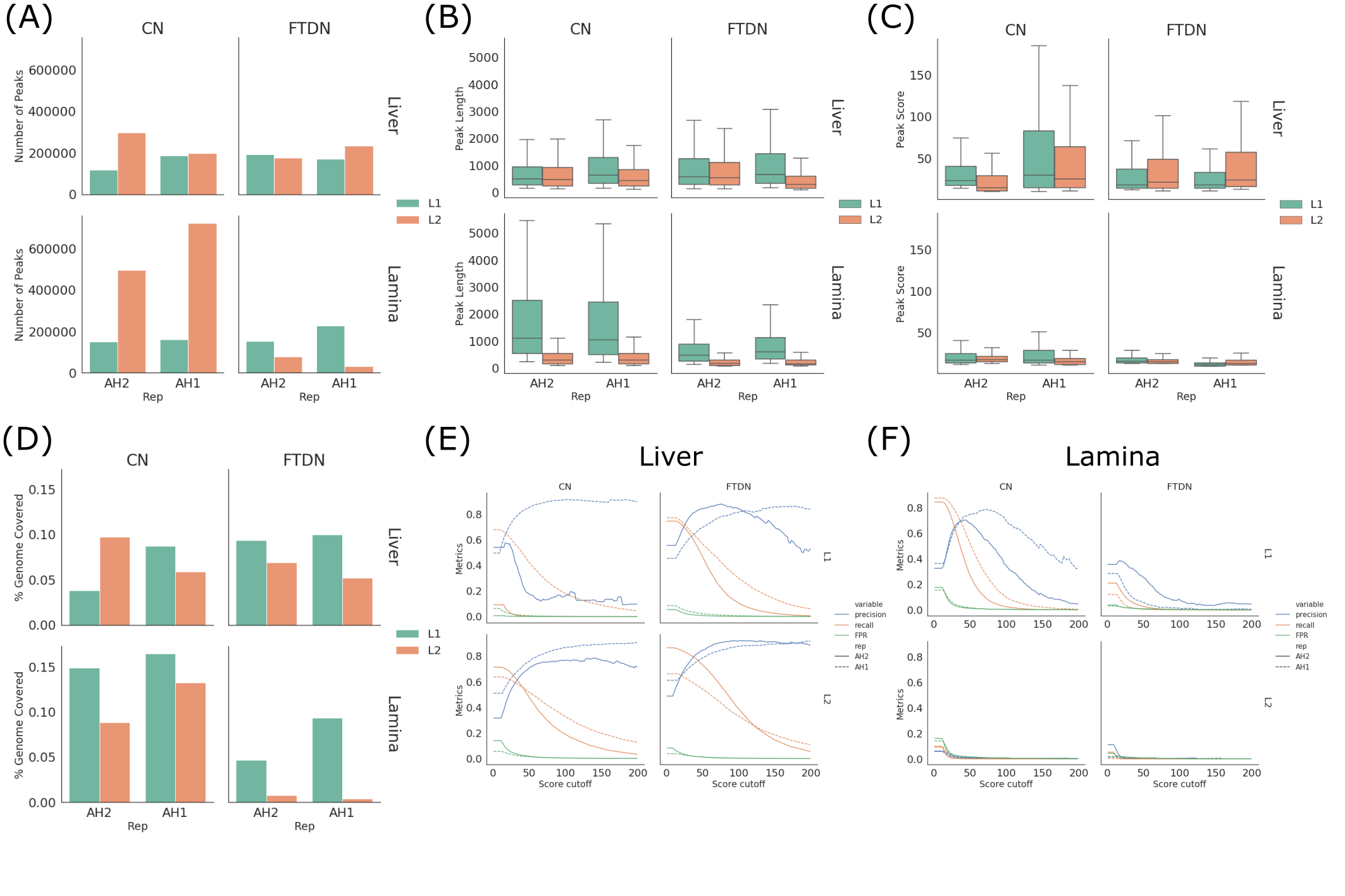

Supplement: Supplementary Figure 4 — MACS2 peak calling statistics. (A) Number of peaks, (B) peak length distribution, (C) peak score distribution, and (D) percent of genome covered by peaks for each library. (E,F) Peak metrics assessed using ChIP-seq dataset in liver (E) and lamina (F) libraries. [file Image_4.TIF]
