# Supplementary material for: Successful ATAC-Seq From Snap-Frozen Equine Tissues
Source: Front Genet. 2021 Jun 16;12:641788. doi: 10.3389/fgene.2021.641788 (PMC8242358; doi:10.3389/fgene.2021.641788)
Supplement: Supplementary file 6 [file Data_Sheet_2.docx]

**Supplementary Methods**

**Nuclei extraction from fresh liver tissue**

1. Weigh tissue and record value (less than 500mg)
2. Mince tissue with razor blade or scissors in polystyrene weighing dish
3. Add proteinase tablet to Sucrose Buffer just prior to use. Transfer minced tissue into a gentle MACS C tube with 10 ml of Sucrose buffer
4. Homogenize tissue using gentle MACS Dissociator Program "E.01c Tube." Some tissues may require homogenizing more than once. Record the number of homogenization steps
5. Filter homogenate using 100 µM Steriflip Vacuum Filter System
6. Bring volume to 9.9 ml with Sucrose buffer. Record volume and amount of sucrose buffer added
7. Add 1.1 ml DMSO to samples (10% final concentration), pipetting several times to adequately mix
8. Set Freezing containers with the appropriate amount of isopropanol. Aliquot into cryotube vials (approximately 1.8 ml per aliquot). Freeze at -80°C overnight in Nalgene Cryo 1°C Freezing Container, then move to -80° C for long-term storage

**Nuclei extraction from fresh lamina tissue**

1. Weigh tissue and record value (less than 300mg)
2. Mince tissue with razor blade or scissors in polystyrene weighing dish
3. Incubate in 25mL collagenase-dispase at 37 degrees while mixing for at least 30 minutes
4. Keep the tissue in collagenase solution while use TissueTearor to begin the homogenization process until a nicely homogenized solution is formed. Incubate for 5 more minutes in collagenase
5. Centrifuge (800g for 5 minutes) and pour off collagenase
6. Add 15mL Hank's Balanced Salt Solution to wash. Centrifuge and pour off again
7. Homogenize tissue using gentle MACS Dissociator Program "E.01c Tube." Some tissues may require homogenizing more than once. Record the number of homogenization steps.
8. Filter homogenate using 100 µM Steriflip Vacuum Filter System
9. Bring volume to 9.9 ml with Sucrose buffer. Record volume and amount of sucrose buffer added
10. Add 1.1 ml DMSO to samples (10% final concentration), pipetting several times to adequately mix
11. Set Freezing containers with the appropriate amount of isopropanol. Aliquot into cryotube vials (approximately 1.8 ml per aliquot). Freeze at -80°C overnight in Nalgene Cryo 1°C Freezing Container, then move to -80° C for long-term storage

**Solutions for nuclei extraction from fresh tissues**

Sucrose Buffer:

| Reagents | Final conc | Volume for 500ml |
| --- | --- | --- |
| 0.5M D-Sucrose | 250 mM | 250 ml |
| 1M MgCl_2_ | 1 mM | 5 ml |
| 10M Tris-HCl pH7.5 | 10 mM | 0.5 ml |
| Sterile Water | NA | Up to 500ml |
| Filter sterilize with 500 mL 0.2 μM Filter System. Store at 4°C. Add Complete Protease  Inhibitor Tablet (1 per 50mL solution) just prior to use. Keep on ice when in use.  Pre-cool centrifuge to 4° C. All centrifugations should be done at 4° C | | |

Type I Collagenase: Worthington Biochemical Corporation

Dispase II: ThermoFisher

Hank’s Balanced Salt Solution: ThermoFisher

**Nuclei extraction from frozen tissues (Lab 1)**

1. Take 100mg of tissue
2. Using a scalpel blade, chop the tissue into small pieces on a dish that is kept on dry-ice
3. Place the tissue pieces in a dounce and allow it to thaw for few minutes on ice
4. Add 1ml 1XHB buffer (with Protease Inhibitor Cocktail) and dounce with loose douncer and then with tight douncer
5. Filter on a 40μm corning cell stainer
6. Rinse the filter with 1ml 1XHB buffer (with Protease Inhibitor Cocktail)
7. Take 400μl of the collected solution and put them in a 2ml “Lo-Bind eppendorf tube”
8. Add 400μl of 50% Iodixanol solution to the 400μl of cell solution (final 25% Iodixanol)
9. Proceed with iodixanol gradient:
   - Slowly add 600μl of 29% Iodixanol solution under the previous mixture
   - Slowly add 600μl of 35% Iodixanol solution under the 29% Iodixanol solution
10. In the swinging bucket centrifuge, centrifuge the 2ml tubes for 20minutes at 3500g
11. A thin “whitish” band should appear between layer 2 and 3 (from the top). Collect this band (200μl) and transfer it to a new collection tube
12. Take an aliquot of 20μl and add trypan blue for nuclei purity evaluation (microscope) and counting
13. Take 50.000 nuclei as determined by nuclei counting and proceed to library preparation

**ATAC library prepareation (Lab 1)**

1. Transfer 50.000 nuclei to a new tube containing 1ml of ATAC-RSB + 0.1%Tween20
2. Centrifuge the nuclei for 10minutes at 500g at 4°C
3. Aspirate supernatant and continue with tagmentation using Tn5 enzyme (Illumina, ref #15027865) following manufacturer recommendations
4. Purify tagmented DNA using Diapure columns (Diagenode, C03040001)
5. Prepare libraries using NEBNext High-Fidelity 2X Master Mix (NEB Cat# M0541) from purified tagmented DNA (starting amount 10µl)
6. Purify and double size select to 100bp-600bp
7. Quantify using Qubit™ dsDNA HS Assay Kit (Thermo Fisher Scientific, Q32854)
8. Analyze fragment size using High Sensitivity NGS Fragment Analysis Kit (DNF-474) on a Fragment Analyzer™ (Advanced Analytical Technologies, Inc.)

**Solutions for nuclei extraction from frozen tissues (Lab 1)**

6XHB Stable buffer:

| Reagents | Final conc | Volume for 100ml |
| --- | --- | --- |
| 1M CaCl2 | 30 mM | 3 ml |
| 1M Mg(Ac)2 | 18 mM | 1.8 ml |
| 1M Tris-HCl pH7.8 | 60 mM | 6 ml |
| Sterile Water | NA | Up to 100ml |

6XHB Unstable buffer (kept in the fridge): 650 µl/sample

| Reagents | Final conc | Volume for 1sample |
| --- | --- | --- |
| 6XHB Stable buffer | 6X | 650 µl |
| PMSF 100mM | 0.1 mM | 0.65 µl |
| B-mercaptoethanol 14.3M | 1 mM | 0.045 µl |

1XHB Unstable buffer (kept in the fridge): 2 ml/sample

| Reagents | Final conc | Volume for 1sample |
| --- | --- | --- |
| 6XHB Unstable buffer | 1X | 333.33 µl |
| Sucrose 1M | 18 mM | 640 µl |
| EDTA 500mM | 60 mM | 0.40 µl |
| IgepalCA630 10% | 0.1% or 0.4% | 20 µl or 80 µl |
| Sterile Water | NA | 1006.27 µl |

50% Iodixanol Solution: 400 µl/sample

| Reagents | Final conc | Volume for 1sample |
| --- | --- | --- |
| 6XHB Unstable buffer | 1X | 66.67 µl |
| Iodixanol stock solution (60%) | 50% | 333.33 µl |

29% Iodixanol Solution: 600 µl/sample

| Reagents | Final conc | Volume for 1sample |
| --- | --- | --- |
| 6XHB Unstable buffer | 1X | 100 µl |
| Sucrose 1M | 160 mM | 96 µl |
| Iodixanol stock solution (60%) | 29% | 290 µl |
| Sterile Water | NA | 114 µl |

35% Iodixanol Solution: 600 µl/sample

| Reagents | Final conc | Volume for 1sample |
| --- | --- | --- |
| 6XHB Unstable buffer | 1X | 100 µl |
| Sucrose 1M | 160 mM | 96 µl |
| Iodixanol stock solution (60%) | 35% | 350 µl |
| Sterile Water | NA | 54 µl |

**ATAC library preparation from frozen tissues (Lab 2)**

The tissue was manually disassociated, isolated nuclei were quantified using a hemocytometer, and 100,000 nuclei were tagmented as previously described (Buenrostro et al. 2013), with some modifications based on (Corces et al. 2017) using the enzyme and buffer provided in the Nextera Library Prep Kit (Illumina). Tagmented DNA was then purified using the MinElute PCR purification kit (Qiagen), amplified with 10 cycles of PCR, and purified using Agencourt AMPure SPRI beads (Beckman Coulter). Resulting material was quantified using the KAPA Library Quantification Kit for Illumina platforms (KAPA Biosystems), and sequenced with PE42 sequencing on the NextSeq 500 sequencer (Illumina).
